# Neurocritical care management supported by multimodal brain monitoring after acute brain injury

**DOI:** 10.5935/2965-2774.20230036-en

**Published:** 2023

**Authors:** Elisabete Monteiro, António Ferreira, Edite Raquel Mendes, Sofia Rocha e Silva, Isabel Maia, Cláudia Camila Dias, Marek Czosnyka, José Artur Paiva, Celeste Dias

**Affiliations:** 1 Department of Intensive Care Medicine, Centro Hospitalar e Universitário São João - Porto, Portugal; 2 Knowledge Management Unit, Department of Community Medicine, Information and Health Decision Sciences, Faculdade de Medicina, Universidade do Porto - Porto, Portugal; 3 Brain Physics Laboratory, Division of Neurosurgery, Department of Clinical Neurosciences, University of Cambridge - Cambrigde, United Kingdom

**Keywords:** Acute brain injury, Autoregulation, Optimal cerebral perfusion pressure, Prognosis, Multimodal brain monitoring, Critical care outcome, Intensive care units

## Abstract

**Objective:**

To evaluate the association between different intensive care units and levels of brain monitoring with outcomes in acute brain injury.

**Methods:**

Patients with traumatic brain injury and subarachnoid hemorrhage admitted to intensive care units were included. Neurocritical care unit management was compared to general intensive care unit management. Patients managed with multimodal brain monitoring and optimal cerebral perfusion pressure were compared with general management patients. A good outcome was defined as a Glasgow outcome scale score of 4 or 5.

**Results:**

Among 389 patients, 237 were admitted to the neurocritical care unit, and 152 were admitted to the general intensive care unit. Neurocritical care unit management patients had a lower risk of poor outcome (OR = 0.228). A subgroup of 69 patients with multimodal brain monitoring (G1) was compared with the remaining patients (G2). In the G1 and G2 groups, 59% *versus* 23% of patients, respectively, had a good outcome at intensive care unit discharge; 64% *versus* 31% had a good outcome at 28 days; 76% *versus* 50% had a good outcome at 3 months (p < 0.001); and 77% *versus* 58% had a good outcome at 6 months (p = 0.005). When outcomes were adjusted by SAPS II severity score, using good outcome as the dependent variable, the results were as follows: for G1 compared to G2, the OR was 4.607 at intensive care unit discharge (p < 0.001), 4.22 at 28 days (p = 0.001), 3.250 at 3 months (p = 0.001) and 2.529 at 6 months (p = 0.006). Patients with optimal cerebral perfusion pressure management (n = 127) had a better outcome at all points of evaluation. Mortality for those patients was significantly lower at 28 days (p = 0.001), 3 months (p < 0.001) and 6 months (p = 0.001).

**Conclusion:**

Multimodal brain monitoring with autoregulation and neurocritical care unit management were associated with better outcomes and should be considered after severe acute brain injury.

## INTRODUCTION

Acute brain injury (ABI) can occur in several different situations, the two most frequent of which are traumatic brain injury (TBI) and subarachnoid hemorrhage (SAH), causing a high socioeconomic burden around the world.^(1,2)^ Several studies suggest that admission to the neurocritical care unit (NCCU) is associated with significantly decreased mortality and increased rates of hospital discharge.^(3,4)^ The presence of a neurointensivist was also associated with improved clinical outcomes, and this effect was more evident in patients with SAH.^(5)^ A global survey of outcomes of neurocritical care patients showed that neurological severity of the illness and the absence of a dedicated NCCU are independent predictors of mortality,^(6)^ favoring the admission of patients with acute brain injury to the NCCU. The primary focus of neurocritical care is the early detection and prevention of secondary brain injury,^(7)^ as the consequences of the primary lesion are often irreversible.^(8)^

Continuous bedside monitoring is crucial for the detection of secondary brain insults. Multimodal brain monitoring (MMM) has been recommended by experts^(9)^ as an important, but non evidence-based, tool to manage severe ABI in intensive care units (ICUs). Multimodal brain monitoring is an evaluation of cerebral function according to multiple modalities in a single patient, providing an integrated interpretation of any secondary insults the patient may undergo. Multimodal brain monitoring should be performed continuously to avoid missing any significant events. Data should be collected simultaneously, time-synchronized and displayed in an integrated fashion,^(8)^ providing targeted individualized care. Ideal MMM should allow simultaneous and continuous bedside assessment of cerebral hemodynamics, oxygenation and metabolism.^(8)^

Multimodal brain monitoring includes variables provided by different devices, including intracranial pressure (ICP), cerebral perfusion pressure (CPP),^(10,11)^ cerebral oximetry by near infrared spectroscopy (NIRS),^(12)^ brain tissue oxygenation (pbtO_2_),^(13)^ cerebral blood flow (CBF) evaluated by transcranial Doppler^(14)^ and/or thermal diffusion flowmetry (CBF-TDF),^(15,16)^ microdialysis,^(17)^ continuous electroencephalography (cEEG)^(18)^ and autoregulation evaluation using the pressure reactivity index (PRx).^(9,19)^

Impaired autoregulation leads to secondary insults and is an independent predictor of fatal outcomes following ABI, specifically TBI.^(3)^ Therefore, the continuous evaluation of autoregulation with the PRx targeting optimal CPP assessment^(20)^ may be an important tool of MMM and is feasible at bedside.^(21,22)^ Despite retrospectively published data about the association between cerebral autoregulation and acute brain injury outcome^(21,23,24)^ suggesting that preserved autoregulation leads to a better prognosis, there is still scarce evidence of the benefits of MMM provided by a dedicated team.

We retrospectively reviewed the clinical files of patients requiring level III ICU admission with spontaneous SAH or TBI. In both illnesses, patients have a high risk of deterioration due to secondary brain damage,^(25)^ and the main objective of management in ICUs is to preclude deterioration.

Our hypothesis is that specialized neurocritical care and MMM together may accomplish that objective and maximize outcomes, namely, quality of life and mortality in patients with ABI.

## METHODS

### Patient selection

We included all patients with severe ABI (spontaneous SAH and TBI) admitted to our Intensive Care Department at *Centro Hospitalar e Universitário São João* between March 2014 and December 2016. The allocation of patients to the general ICU (GICU) occurred due to a shortage of bed availability in the NCCU. A total of 389 patients were enrolled in this study. Patients less than 18 years old, pregnant females and those with an expected survival of less than three days were excluded. The local Research Ethics Committee approved the protocol and data collection.

### Data collection

Patient files were retrospectively reviewed, and demographic and clinical variables, such as age, sex, and Glasgow coma scale (GCS) at first aid and at hospital admission, were recorded. Disease severity and mortality prediction on admission were calculated using the Simplified Acute Physiology Score II (SAPS II).^(26)^ Regarding systemic monitoring, all patients had a Philips IntelliVue® multiparameter monitor that allowed bedside continuous acquisition of electrocardiogram, heart rate, respiratory rate, arterial blood pressure (ABP), pulse oximetry and end-tidal carbon dioxide. Regarding MMM^(27)^ performed in the NCCU, the following variables were included: ABP, ICP, CPP, optimal CPP (CPPopt), NIRS, pbtO_2_, CBF and PRx for continuous evaluation of autoregulation, calculated as a moving Pearson correlation between the slow waves of ICP and ABP. Calculation of CPPopt and continuous data recording was performed with the software ICM + ®, (http://www.neurosurg.cam.ac.uk/icmplus).^(28)^ In the GICU, patients were monitored using only ABP, ICP, and CPP with or without NIRS (depending on clinical decision), and data were documented manually in the clinical records.

Outcomes at ICU discharge, 28 days, 3 months and 6 months were assessed with the Glasgow outcome scale (GOS)^(23)^ where a bad outcome was defined as GOS 1, 2 or 3 and a good outcome was defined as GOS 4 or 5.

**Figure 1:**
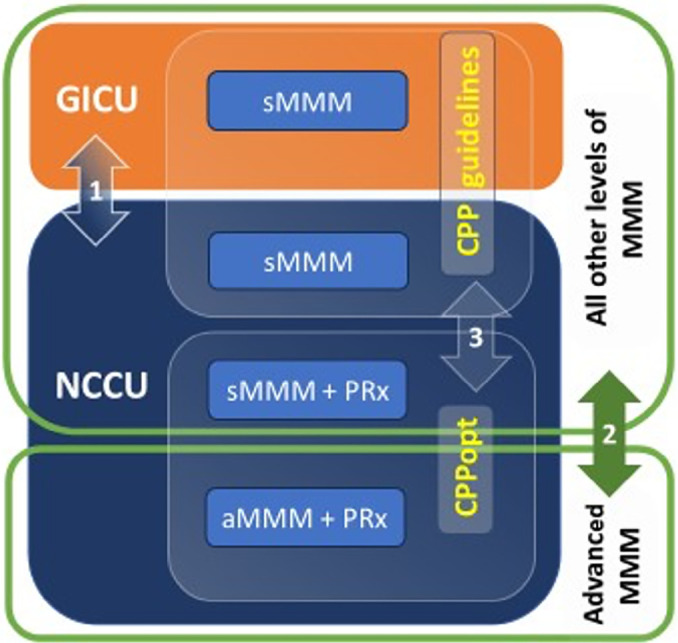
Schematic representation of analysis performed in different intensive care units with different levels of multimodal monitoring.

We performed a three-step analysis based on the level of monitoring and type of ICU, as shown in figure 1.

GICU - general intensive care unit; NCCU - neurocritical care unit; sMMM - standard multimodal monitoring (includes intracranial pressure, cerebral perfusion pressure and cerebral oximetry with near infrared spectroscopy); sMMM+PRx - standard multimodal monitoring autoregulation evaluation combined with pressure reactivity index; aMMM+PRx - advanced multimodal monitoring (sMMM+PRx combined with brain tissue oxygenation with Clarke-type probe and cerebral blood flow with thermal diffusion probe); CPPopt - patients managed according to autoregulation with individualized bedside optimal cerebral perfusion pressure; CPP guidelines - patients managed with cerebral perfusion pressure indicated by guidelines;

In the first analysis, we compared the two different types of ICU management (NCCU and GICU). Second, we compared patients managed with MMM, including ABP, ICP, CPP, NIRS, pbtO_2_, CBF and PRx, against the patients managed with standard monitoring (either in the NCCU or GICU). Third, we compared the subgroup of patients managed with CPPopt-guided therapy in the NCCU against the patients managed according to guidelines.^(29)^

### Statistical methods

Continuous variables are expressed as the mean ± standard deviation (SD) or medians and interquartile range (IQR). Categorical variables are presented as counts (n) or percentages (%). The GOS results were dichotomized into bad outcomes (≤ 3) and good outcomes (> 3), and a comparison analysis was performed for all patients. For continuous variables, nonparametric Mann‒Whitney or Kruskal-Wallis tests were used as appropriate, according to normality assumptions and the number of groups compared. For categorical variables, a chi-square test and Fisher’s exact test were used, as appropriate. To obtain a more thorough understanding of the factors associated with poor outcomes and mortality (dependent variables), univariate and multivariate logistic regression modeling was performed, with sex, age, GCS at first aid and ICU type as independent variables.

The time elapsed from admission to the ICU to mortality (length of stay in the ICU) was evaluated using survival analysis. The cumulative probabilities of event-free survival were estimated using the Kaplan-Meier method, and the LogRank and Breslow tests were used to compare groups according to monitoring level.

The significance level used was 0.05. Statistical analysis was performed using the software Statistical Package for the Social Sciences, version 24.

## RESULTS

### First analysis: neurocritical care unit *versus* general intensive care unit management

The studied population consisted of 389 patients, of whom 237 (61%) were admitted to the NCCU and 152 (42%) to the GICU, with a median age of 60 (46 - 75) years in the NCCU group and 63 (48 - 75) years in the GICU group. Regarding sex, 259 patients were male (67%), of whom 150 were in the NCCU (58%) and 109 were in the GICU (42%).

SAPS II also showed a significant difference between ICUs, with a median score of 40 in the NCCU group and 47 in the GICU group (p < 0.001). The GCS evaluated at the local first aid was 12 for the NCCU group and 9 for the GICU group (p = 0.013). There were no differences between ICUs regarding length of stay (LOS) in the ICU. The median hospital LOS was 30 days (19 - 54) for the NCCU group and 28 days for the GICU group (15 - 46).

The proportion of good outcomes was significantly different (p < 0.001) for the two types of ICUs (NCCU and GICU, respectively) at ICU discharge (43% *versus* 10%), 28 days (50% *versus* 20%), 3 months (72% *versus* 37%) and 6 months (80% *versus* 43%) (Table 1).

**Table 1:** Demographic, clinical, outcome and survival data in the neurocritical care unit and general intensive care unit

	NCCU (n = 237)	GICU (n =152)	p value^*^
Sex, male	150 (63)	109 (72)	0.086†
Age	60 (46 - 75)	63 (48 - 75)	0.852
LOS in ICU	15 (8 - 25)	13 (8 - 21)	0.116
LOS in hospital	30 (19 - 54)	28 (15 - 46)	0.020
SAPS II	40 (29 - 50)	47 (40 - 57)	< 0.001
SAPS II mortality, %	25 (10 - 46)	47 (40 - 57)	< 0001
GCS at first aid	12 (8 - 14)	10 (6 - 14)	0.013
GCS at hospital	9 (4 - 13)	3 (3 - 10)	< 0.001
Good outcome‡			
ICU	102 (43)	15 (10)	< 0.001†
28 days	99 (50)	27(20)	< 0.001†
3 months	120 (72)	47 (37)	< 0.001†
6 months	124 (80)	53 (43)	<0.001†
Mortality§			
ICU	28 (11)	37 (25)	0.001†
28 days	15 (8)	43 (31)	< 0.001†
3 months	13 (8)	49 (38)	< 0.001†
6 months	11 (7)	50 (35)	< 0.001†

NCCU - neurocritical care unit; GICU - general intensive care unit; LOS - length of stay; ICU - intensive care unit; SAPS II - Simplified Acute Physiology Score; GCS - Glasgow coma scale.^*^ Mann-Whitney test; † chi-square test; ‡ good outcome: Glasgow outcome scale 4 and 5; § mortality: Glasgow outcome scale = 1. Results expressed as n (%) or median (P25-P75 percentile).

Logistic regression was performed to compare outcomes and mortality rates for both ICUs. After adjusting outcomes and mortality rates for age, sex, GCS at first aid and SAPS II, patients managed at the NCCU still presented a lower risk of having a bad outcome (OR = 0.228 [0.112 - 0.466]) when compared to patients managed at GICUs.

### Second analysis: multimodal brain monitoring in the neurocritical care unit *versus* standard monitoring in either the neurocritical care unit or general intensive care unit management

We compared the subgroup of patients who received MMM, including ICP, CPP, NIRS, pbtO2, CBF and CPPopt-guided therapy, in the NCCU, designated as G1 (69 patients), with the remaining 320 patients (G2) admitted either to the GICU or NCCU. The two groups showed no differences regarding sex, ICU or hospital LOS. The median (P25 - P75) GCS at hospital admission was 4 (3 - 12) for G1 and 8 (3 - 13) for G2 (p = 0.05), and the SAPS II score was 40 (29 - 49) for G1 and 43 (33 -55) for G2 (p = 0.047).

Regarding outcomes, G1 patients had a good outcome: 59% of at ICU discharge, 64% at 28 days, and 76% at 3 months. The G2 patients had a good outcome: 23% of at ICU discharge, 31% at 28 days and 50% at 3 months (p < 0.001 at all 3 time points, comparing G1 *versus* G2). At 6 months, the proportion of patients with a good outcome was 77% in G1 and 58% in G2 (p = 0.005).

Mortality rates were 7% for G1 and 19% for G2 at ICU discharge (p = 0.02), 7% for G1 and 20% for G2 at 28 days (p = 0.013), 9% for G1 and 25% for G2 at 3 months (p = 0.008) and 13% for G1 and 25% for G2 at 6 months (p = 0.039).

When adjusting outcome for age, in a multivariate analysis and using good outcome as the dependent variable, the results were as follows for G1 compared to G2: the OR was 4.607 (2.666 - 7.962) at ICU discharge (p < 0.001), 4.226 (2.409 - 7.413) at 28 days (p = 0.001), 3.250 (1.719 - 6.144) at 3 months (p = 0.001) and 2.529 (1.310 - 4.882) at 6 months (p = 0.006).

Differences between G1 and G2 regarding good outcome remained when adjusted for severity. Regarding mortality, when adjusted for SAPS II, there were no statistically significant differences between the groups (Table 2).

**Table 2:** Demographic, clinical, and outcome data according to monitoring level

	GICU and standard MMM (n = 152)	NCCU and standard MMM (n = 110)	NCCU and standard MMM with PRx (n = 58)	NCCU and advanced MMM with PRx (n = 69)	p value^*^
Sex, male	109 (72)	71 (65)	34 (59)	45 (65)	
Age	63 (48 - 75)	65 (50 - 77)	60 (45 - 74)	58 (41 - 69)	0.255
ICU LOS	13 (8 - 21)	10 (6 - 18)	21 (11 - 30)	23 (25 - 29)	<0.001
Hospital LOS	28 (15 - 46)	24 (17 - 44)	33 (19 - 67)	41 (26 - 67)	< 0.001
SAPS II	47 (40 - 57)	39 (24 - 51)	43 (34 - 51)	40 (29 - 49)	< 0.001
SAPS II mortality, %	39 (25 - 62)	23 (6 - 48)	31 (18 - 48)	25 (11 - 44)	< 0.001
GCS at first aid	9 (6 - 14)	13 (10 - 15)	11 (7 - 14)	10 (6 - 14)	< 0.001
GCS at hospital	6 (3 - 10)	11 (7 - 14)	8 (6 - 12)	4 (3 - 12)	< 0.001
Good outcome‡					
ICU	15 (10)	52 (47)	9 (16)	41 (59)	< 0.001†
28 days	27 (20)	46 (54)	8 (19)	45 (65)	< 0.001†
3 months	47 (37)	51 (75)	19 (58)	50 (77)	< 0.001†
6 months	53 (43)	54 (86)	20 (69)	50 (78)	< 0.001†
Mortality§					
ICU	37 (24)	12 (11)	11 (19)	5 (7)	0.003†
28 days	43 (31)	6 (7)	4 (9)	5 (7)	< 0.001†
3 months	49 (38)	4 (6)	3 (9)	6 (9)	< 0.001†
6 months	50 (41)	1 (2)	2 (7)	8 (12)	< 0.001†

GICU - general intensive care unit; MMM - standard multimodal; NCCU - neurocritical care unit; PRx - pressure reactivity index; ICU - intensive care unit; LOS - length of stay; SAPS II - Simplified Acute Physiology Score; GCS - Glasgow coma scale. Good outcome includes Glasgow outcome scale 4 and 5.

* Kruskal-Wallis test; † chi-square test; ‡ bad outcome: Glasgow outcome scale between 1 and 3; § mortality: Glasgow outcome scale = 1. Results expressed as n (%) or median (P25-P75 percentile).

### Third analysis: optimal cerebral perfusion pressure guided therapy management *versus* guidelines management

We compared patients managed at the NCCU with CPPopt-guided therapy (n = 127) against patients managed according to the guidelines (n = 262). The group managed according to CPPopt, evaluated with the PRx, showed better outcomes and mortality rates when compared to patients managed according to the guidelines. The proportion of good outcomes in the two groups was, respectively, 39.4% *versus* 25.7% at ICU discharge (p = 0.006), 47.3% *versus* 32.7% at 28 days (p = 0.009), 70.4% *versus* 50% at 3 months (p = 0.001) and 75.3% *versus* 52.5% at 6 months (p = 0.004).

**Figure 2:**
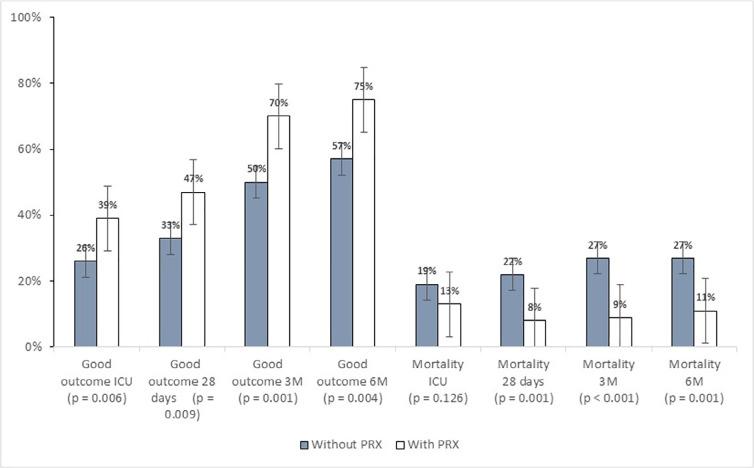
Patient management with or without optimal cerebral perfusion pressure guided therapy: outcomes and mortality.

Mortality was lower in the group managed with the CPPopt protocol: 92% *versus* 78% (p = 0.001) at 28 days, 90.8% *versus* 73% (p < 0.001) at 3 months and 89.2% *versus* 72.6% (p = 0.001) at 6 months (Figure 2).

Good outcomes included Glasgow outcome scale scores of 4 and 5, and poor outcomes included Glasgow outcome scale scores of 1, 2 and 3. Mortality: Glasgow outcome scale = 1.

ICU - intensive care unit; PRx - pressure reactivity index.

## DISCUSSION

In this retrospective single-center study, we focused on the differences between ICU organization and management of acute brain injury with distinct levels of neuromonitoring and its relationship to outcome, specifically bad or good outcomes dichotomized by GOS and mortality rate (GOS 1).

The main findings in our study include the following: (1) NCCU team organization centered in acute brain injury management appears to be associated with better results than general ICU management, independent of the level of MMM; (2) patients managed in the NCCU with MMM seem to have better outcomes; and (3) neuromonitoring complemented with bedside evaluation of autoregulation with PRx and CPPopt-guided therapy management by a dedicated NCCU team provided the best outcomes.

In the first analysis, we highlighted the importance of ICU type and the finding that not only survival but also 6 months good outcome were better in patients managed at NCCU than in those managed in the GICU, with statistical significance (p < 0.001). Our findings are corroborated by the published literature,^(30)^ which stresses that a very well-trained multidisciplinary team centered on the neurocritical patient is crucial for the prompt detection of changes in neuromonitoring and adequate correction, both of which are essential to avoid secondary injury and achieve a better prognosis. Currently, the role of the NCCU in the management of critically ill patients with acute brain injury is recommended by experts (strong recommendation, moderate quality of evidence).^(9)^ In patients after aneurysmal subarachnoid hemorrhage, the recommendation is also that they should be treated at high-volume centers (moderate quality evidence-strong recommendation).^(27)^ These high-volume centers have many features that may contribute to improved outcomes, such as neurointensive care units run by neurointensivists.^(6)^

Second, by comparing the group of patients managed in the NCCU with ABP, ICP, CPP, NIRS, pbtO_2_, CBF, PRx and CPPopt-guided therapy (69 patients) with the remaining 320 patients, we found evidence that optimal CPP-guided therapy with MMM enriched with oxygen and flow variables, such as pbtO_2_ and CBF-TDF, achieved better outcomes. Those patients in the NCCU had a significantly higher chance of having a good outcome than the remaining patients. Because mortality adjusted for age and severity was not significantly different, we may infer that the contribution to poor outcomes mainly results from GOS-2 and GOS-3 in patients managed without complete monitoring (ABP, ICP, CPP, NIRS, pbtO_2_, CBF, PRx and CPPopt-guided therapy). Bouzat et al. showed that the level of brain neuromonitoring offered and the increase in accuracy provided by advanced MMM to detect cerebral hypoperfusion and hypoxia have an impact on outcome and mortality^(31)^ in favor of its use.

Finally, we underline the importance of individualized treatment of ABI patients using CPPopt with real-time evaluation of autoregulation using the PRx, since the outcome results at all assessment time points favored this methodology, even after adjustment for severity, as shown in figure 3. Several studies have shown that targeted individual CPP management at the bedside using cerebrovascular pressure reactivity is feasible, and a large deviation from CPPopt seems to be associated with adverse outcomes.^(32)^ In TBI patients, Aries et al.^(33)^ showed that patients with a median CPP close to CPPopt were more likely to have a favorable outcome than those in whom median CPP widely deviated from CPPopt. Deviations from individualized CPPopt were more predictive of outcome than deviations from the CPP recommended by the guidelines. In severe SAH, the calculation of CPPopt is also possible, and an actual CPP below CPPopt is associated with low CBF.^(34)^ This information may provide important clues regarding long-term outcomes since, as Rasulo showed, a PRx above the 0.2 threshold and a CPP below the CPPopt range are associated with worse outcome.^(20)^

### Limitations

Data were collected retrospectively at a single medical center; the time course for the study was only 22 months; and the selected patients included acute brain injury patients with TBI and SAH but excluded those with intracerebral hemorrhage.

Another major limitation is the selection of patients with chances of survival.

SAPS II was used as a severity index but does not contain any neurological variables besides GCS, and perhaps it is not sufficiently sensitive for this heterogeneous population.

Another limitation is that patients were not randomly allocated to the different care environments, and care providers were not blinded to monitoring interventions. This may not be able to be fully corrected by multivariate analysis.

This study may also have a bias of bed selection and availability since there is a possibility that beds were made available depending on the potential survivability of the patient. This is supported by the SAPS II score and GCS differences between the NCCU and GICU patients.

Finally, despite data collection at a high-volume center, these results may benefit from prospective research and extension to multicenter studies, whereby further validation is warranted.

## CONCLUSION

Brain multimodal monitoring, including intracranial pressure, cerebral perfusion pressure, brain oximetry and oxygenation and cerebral blood flow complemented with continuous bedside assessment of autoregulation and individualized optimal cerebral perfusion pressure guided therapy in a neurocritical care unit environment, showed better outcomes in severe acute brain injury management.
